# Cancer-Specifically Re-Spliced TSG101 mRNA Promotes Invasion and Metastasis of Nasopharyngeal Carcinoma

**DOI:** 10.3390/ijms20030773

**Published:** 2019-02-12

**Authors:** Huey-Huey Chua, Toshiki Kameyama, Akila Mayeda, Te-Huei Yeh

**Affiliations:** 1 Department of Pediatrics, National Taiwan University Hospital, College of Medicine, National Taiwan University, Taipei 10051, Taiwan; hueyhueychua@gmail.com; 2 Division of Gene Expression Mechanism, Institute for Comprehensive Medical Science, Fujita Health University, Kutsukake-cho, Toyoake, Aichi 470-1192, Japan; tkame@fujita-hu.ac.jp (T.K.); mayeda@fujita-hu.ac.jp (A.M.); 3 Department of Otolaryngology, National Taiwan University Hospital, College of Medicine, National Taiwan University, Taipei 10051, Taiwan

**Keywords:** nasopharyngeal carcinoma, TSG101, TSG101∆154-1054, re-splicing, invasion, metastasis

## Abstract

*TSG101* (Tumor susceptibility 101) gene and its aberrantly spliced isoform, termed TSG101∆154-1054, are tightly linked to tumorigenesis in various cancers. The aberrant TSG101∆154-1054 mRNA is generated from cancer-specific re-splicing of mature TSG101 mRNA. The TSG101∆154-1054 protein protects the full-length TSG101 protein from ubiquitin-mediated degradation, implicating TSG101∆154-1054 protein in the progression of cancer. Here, we confirmed that the presence of TSG101∆154-1054 mRNA indeed caused an accumulation of the TSG101 protein in biopsies of human nasopharyngeal carcinoma (NPC), which was recapitulated by the overexpression of TSG101∆154-1054 in the NPC cell line TW01. We demonstrate the potential function of the TSG101∆154-1054 protein in the malignancy of human NPC with scratch-wound healing and transwell invasion assays. By increasing the stability of the TSG101 protein, TSG101∆154-1054 specifically enhanced TSG101-mediated TW01 cell migration and invasion, suggesting the involvement in NPC metastasis *in vivo*. This finding sheds light on the functional significance of TSG101∆154-1054 generation *via* re-splicing of TSG101 mRNA in NPC metastasis and hints at its potential importance as a therapeutic target.

## 1. Introduction

The multi-complex machinery, termed the ‘endosomal sorting complex required for transport’ (ESCRT), regulates the sorting of ubiquitinated proteins to endosomes, facilitating receptor traffic and turnover, and it has been implicated in normal development, cell differentiation, and growth, as well as the budding of certain enveloped viruses (reviewed in [1,2]). The dysregulation of ESCRT proteins occurs in the development of various human diseases, including many types of cancers and neurodegenerative disorders (reviewed in [3,4]). The ESCRT-I complex has multiple cellular functions, including the regulation of transcription, protein sorting, biogenesis of multi-vesicular bodies, and viral budding (reviewed in [1,5,6]). Although *TSG101* was initially found in a screen for potential tumor-suppressor genes in mouse [7], this product is one component of the ESCRT-I complex. TSG101 knockout mice are embryonically lethal, suggesting that TSG101 is essential for the proliferation and survival of embryonic tissues [8,9]. TSG101 deficiency in primary embryonic fibroblasts and tumor cell lines causes cell cycle arrest at the G1/S transition [10,11]. In addition, TSG101 depletion in tumor cells reduces migration, clonogenicity, and drug-resistance [11,12]. We demonstrated previously that TSG101 contributes to Rta-mediated late gene activation in the productive lytic cycle of Epstein Barr virus, a DNA virus that is implicated in nasopharyngeal carcinoma (NPC) [13].

Malignant tumors often develop a stage-dependent dysregulation of alternative splicing programs and the resulting aberrantly spliced mRNAs are strongly correlated with neoplastic changes, invasion, and poor clinical prognosis (reviewed in [14]). *TSG101* is an established cancer-associated gene and aberrantly spliced TSG101 mRNAs have been reported in various kinds of cancers (reviewed in [15,16]). Besides normal full-length TSG101 mRNA, truncated aberrant mRNA isoforms were found in various cancerous tissues [17,18,19,20,21,22,23,24,25,26], in which genomic mutations of TSG101 are rarely found [20,22,27]. Among the various aberrantly spliced TSG101 mRNAs, an isoform missing internal 901 nucleotides (termed TSG101∆154-1054 or TSG101∆190-1090, which lacks residues 204 to 1104 according to the latest RefSeq NM_006292.3) is predominant in most tumor tissues [17,18,19,21,24]. It is clear from sequencing data that the processing of TSG101∆154-1054 mRNA is due to exon skipping through the inappropriate recognition of weak alternative 5′ and 3′ splice sites in the TSG101 coding exons [24,28]. Our discovery of the mature TSG101 mRNA re-splicing pathway explains the activation of the distant weak alternative splice sites well, since the prior normal splicing events remove all strong competitive authentic splice sites and brings the weak splice sites into close proximity [29].

Detailed examination of TSG101∆154-1054 in pre-neoplastic lesions, as well as biopsies of cervical cancer, revealed a significant correlation between the expression of this transcript and neoplastic progression [24]. In addition, the TSG101∆154-1054 transcript is often present in late-stage breast cancer and it correlates significantly with advanced axillary lymph node metastasis [30]. Importantly, we have recently demonstrated the function of the truncated TSG101∆154-1054 protein generated *via* re-splicing of TSG101 mRNA, i.e., the protection of full-length TSG101 protein from its ubiquitin-mediated proteasomal degradation [31]. Because of the common occurrence of increased TSG101 protein and its splice variant TSG101∆154-1054 in breast tumor progression, here we investigated their potential involvement in the tumorigenesis of NPC.

## 2. Results

### 2.1. TSG101 Pre-mRNA Is Aberrantly Spliced in Nasopharyngeal Carcinoma Tissues from Patients

**Figure 1:**
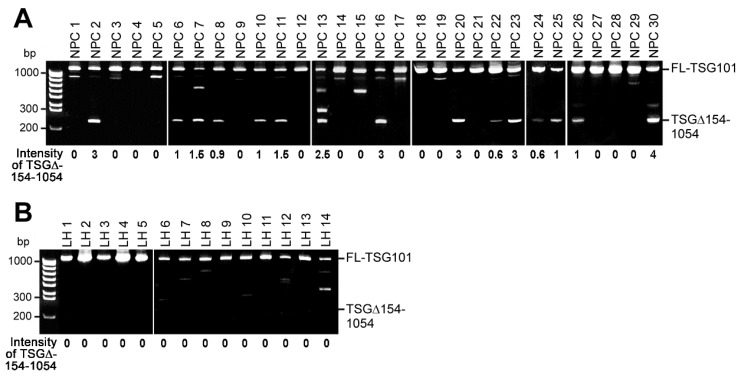
The TSG∆154-1054 mRNA variant is detected predominantly in nasopharyngeal carcinoma (NPC) but not in normal lymphoid hyperplasia (LH). (**A**,**B**) RT-nested-PCR detection of constitutively spliced full-length TSG101 mRNA (FL-TSG101) and aberrantly spliced TSG101 mRNA (TSG∆154-1054) in biopsies of NPC and control LH. The RT-nested-PCR products were analyzed by agarose gel electrophoresis and the quantified intensity of the TSG∆154-1054 mRNA variant is indicated.

Using reverse-transcription, followed by nested polymerase chain reaction (RT-nested-PCR), a shortest isoform (around 250-bp marker) among the various TSG101 isoforms was most frequently observed besides the full-length TSG101 transcripts in almost half of the NPC tissues (18 of 38 cases; 30 cases are shown in Figure 1A). Sequence analysis of this isoform revealed that it is the well-documented cancer-associated aberrantly spliced TSG101 isoform, the so-called TSG101∆154-1054 (abbreviated as TSG∆154-1054 hereafter). In contrast, this TSG∆154-1054 mRNA was rarely found in non-cancerous lymphoid hyperplasia (LH; 3 of 30 cases; 14 cases are shown in Figure 1B). The difference between NPC tissues and LH tissues is significant (chi-square test; *p* < 0.005).

These results confirmed that cancer-specific aberrant TSG∆154-1054 mRNA is also a common and unique feature in NPC patients.

### 2.2. TSG∆154-1054 Expression Augments the Protein Levels of TSG101

**Figure 2:**
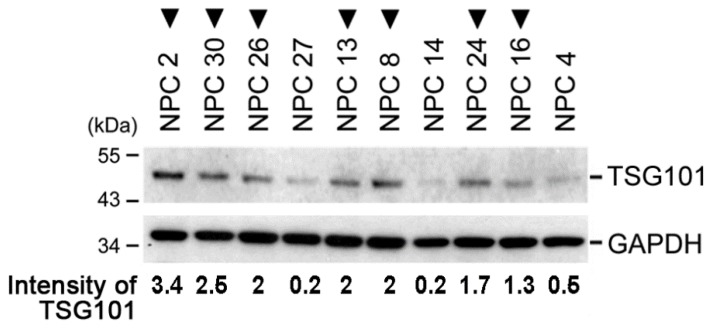
The expression of TSG∆154-1054 correlates with an increased amount of TSG101 protein. The levels of endogenous TSG101 protein expressed in NPC biopsies (see Figure 1A) were measured by immunoblotting. Inverted triangles denote the cases that are positive for TSG∆154-1054 expression. The quantified intensity of the TSG101 protein relative to GAPDH is indicated.

**Figure 3:**
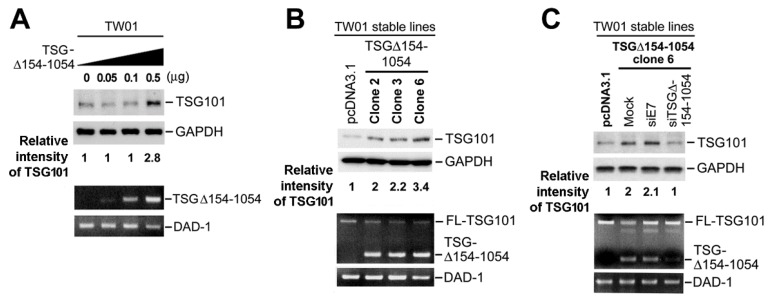
The ectopic expression of TSG∆154-1054 increased levels of TSG101 protein. (**A**) Expression of TSG101 protein in TW01 cells transfected transiently with TSG∆154-1054 plasmid. **Upper panel:** Protein lysates were analyzed by immunoblotting with anti-TSG101 and anti-GAPDH antibodies (control). The quantified intensity of TSG101 protein relative to ‘0 µg’ TSG∆154-1054 in the left lane is indicated, after normalizing with the intensity of the corresponding GAPDH internal control. **Lower panel:** Total RNAs were analyzed by RT-nested-PCR to detect TSG∆154-1054 mRNA. An RT-PCR detecting DAD-1 was performed as a control to verify equal input of cDNA templates. (**B**) Expression of TSG101 protein in TW01 cells (clones 2, 3, 6) stably expressing TSG∆154-1054 and cells expressing empty pcDNA3.1 vector as control. **Upper panel:** Immunoblotting to analyze TSG101 protein. The quantified intensity of TSG101 protein relative to control pcDNA3.1 is indicated after standardizing with the intensity of control GAPDH. **Lower panel:** RT-nested-PCR to analyze FL-TSG101 and TSG∆154-1054 mRNAs with DAD-1 as control. (**C**) Effect of TSG∆154-1054 knockdown on the expression levels of TSG101 mRNA and protein. siTSG∆154-1054 is the targeted siRNA and siE7 is an irrelevant siRNA control. Immunoblotting and RT-nested-PCR were performed as in panels (**A**,**B**).

In ten NPC tissue samples of TSG∆154-1054 positive cases (Figure 2, inverted triangles), protein blotting revealed that they had significantly higher amounts of TSG101 protein than TSG∆154-1054 negative cases (Mann–Whitney–Wilcoxon rank sum test; *p* = 0.015). To verify this finding, a plasmid encoding TSG∆154-1054 was constructed and used for transient transfection assays in an NPC cell line, TW01. Mirroring the finding from NPC biopsies, the amount of endogenous TSG101 protein in TW01 cells increased with the expression of TSG∆154-1054 (Figure 3A). The stable expression of TSG∆154-1054 in TW01 cells also significantly increased the amount of TSG101 protein, but notably, the level of full-length TSG101 mRNA remained constant (Figure 3B). More importantly, the rapid clearance of TSG∆154-1054 by a TSG∆154-1054-specific siRNA (siTSG∆154-1054) caused a reduction of the TSG101 protein (Figure 3C).

Together, these data indicate that the aberrant TSG∆154-1054 mRNA upregulates the full-length TSG101 protein without increasing its mRNA revels.

### 2.3. TSG∆154-1054 Induces TSG101 Protein Stabilization

**Figure 4:**
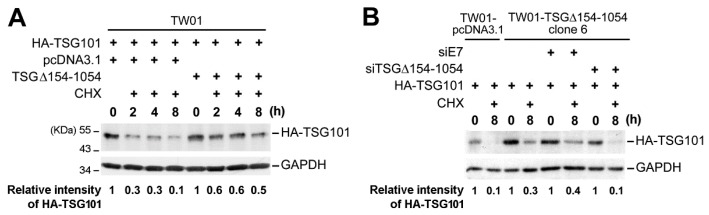
The expression of TSG∆154-1054 promotes stabilization of TSG101 protein. (**A**) Cycloheximide chase assay followed by TSG∆154-1054 expression in TW01 cells to examine stability of TSG101 protein. Immunoblotting was performed to observe the degradation of HA-TSG101 protein during the time course of cycloheximide (CHX) treatment. The quantified intensity of HA-TSG101 protein relative to ‘time 0’ is indicated, after normalizing with the intensity of its corresponding GAPDH internal control. (**B**) The effect of TSG∆154-1054 knockdown on the stability of HA-TSG101 protein. Cycloheximide chase assay with a TW01-TSG∆154-1054 (clone 6) and TW01-pcDNA3.1 (control) stable lines. Quantification was performed as described above.

To perform a kinetic analysis in estimating the turnover of TSG101 by the inhibition of *de novo* synthesis, we used a cycloheximide chase assay. This suggested that the ectopic expression of TSG∆154-1054 markedly stabilize the TSG101 protein after cycloheximide treatment (Figure 4A). To confirm this observation, we specifically repressed TSG∆154-1054 expression using specific siRNA (siTSG∆154-1054) and checked the TSG101 protein levels. Rapid clearance of TSG∆154-1054 in a TSG∆154-1054-expressing stable cell line abrogated TSG101 stabilization after cycloheximide treatment (Figure 4B).

We conclude that the over-expression of TSG∆154-1054 prohibits the degradation of the full-length TSG101 protein, which is fully consistent with our recent discovery of the TSG∆154-1054 protein function as a competitive inhibitor of the ubiquitin-proteasome degradation of TSG101 protein [31]. 

### 2.4. TSG∆154-1054 Increases the TSG101-Dependent Metastatic Activity of Tumor Cells

**Figure 5:**
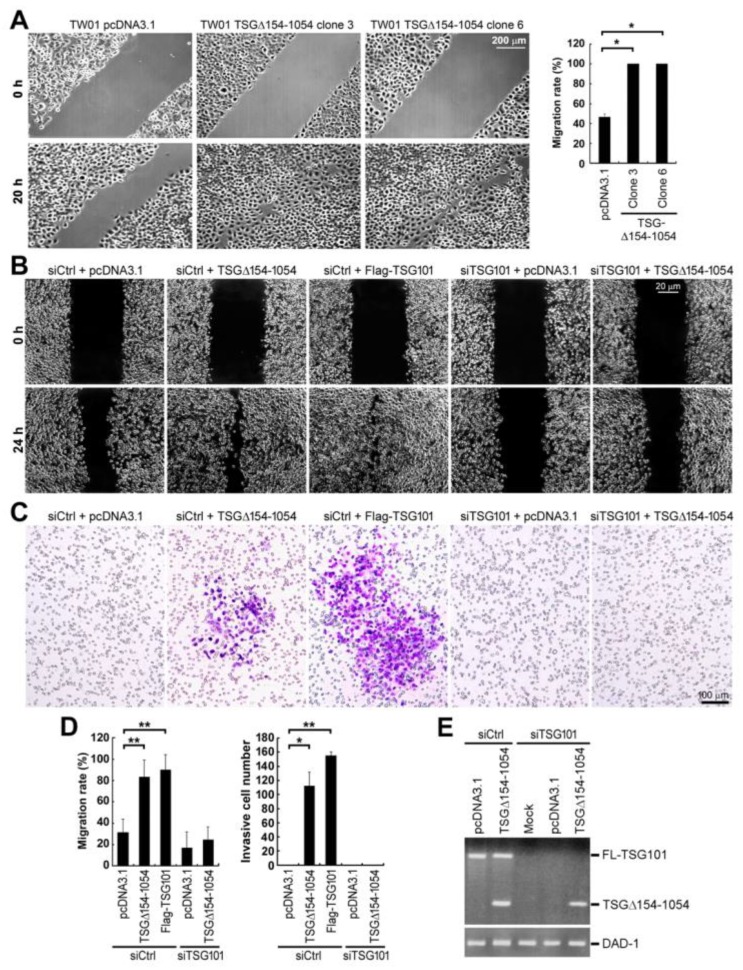
TSG∆154-1054 enhances cell migration and invasion. (**A**) The migration of the TSG∆154-1054 (clones 3, 6) and pcDNA3.1 (control) stable cell lines was examined by a scratch wound-healing assay. Light microscope images at 0 h and 20 h post-scratching are shown. Each image is a representative result from three independent experiments that were performed in duplicate (mean ± SD, * *p* < 0.005 using a two-tailed Student *t* test). (**B**) The effect of TSG∆154-1054 expression with or without TSG101 on cell migratory potential was examined. TW01 cells that were seeded on six-well plates were transfected twice, 24 h apart, with TSG101-targeted siRNA (siTSG101) or control siRNA (siCtrl), and then transfected once with plasmids expressing TSG∆154-1054 and Flag-TSG101 or with control plasmid (pcDNA3.1). These transfected cells were reseeded on 12-well plates to perform a scratch wound-healing assay. (**C**) A transwell invasion assay was performed on the transfected TW01 cells, as described in (**B**). A representative image from three repeated experiments is shown. Purple cells (stained with Giemsa solution) are invading cells, which were counted for the quantification. Dots cluttered backgrounds are porous transwell membranes. (**D**) Quantification of the migratory and invasive potential of the indicated transfected TW01 cells. The migratory rate and the invasion ability were calculated as described in Materials and Methods. The results are presented as mean ± SD, * *p* < 0.0005 and ** *p* < 0.00005 by two-tailed Student *t* test. (**E**) A successful knockdown of TSG101 by siTSG101 and the ectopic expression of TSG∆154-1054 were checked by RT-nested PCR. RT-PCR targeting DAD-1 was performed as a control to verify equal input of cDNA templates.

Since the TSG101 protein has a critical role in cell clonogenicity and migration [11], we next investigated the effects of TSG∆154-1054 expression in tumor cell migration by wound healing assay examined by bright-field microscopy (Figure 5A,B). The migratory ability of the stable TW01 cells expressing TSG∆154-1054 was significantly increased from ~48% to 100% in 20 h (Figure 5A). The mechanism of action by TSG∆154-1054 was achieved through TSG101 stabilization, since TSG∆154-1054 failed to promote tumor cell migration once the cellular TSG101 protein was depleted by TSG101-specific siRNA (Figure 5B,D left graph).

Subsequently, we checked the invasive capacity of these cells by a transwell invasion assay. The ectopic expression of TSG∆154-1054, as well as TSG101, induced the invasiveness of TW01 cells through Matrigel-coated membranes (Figure 5C,D right graph). However, the depletion of TSG101 by TSG101-specific siRNA (siTSG101) fully reduced the invasive potential of TSG∆154-1054-expressing cells in parallel with a loss of migration (Figure 5B–D). The full depletion of TSG101 and the expression of TSG∆154-1054 were clearly verified by RT-nested PCRs, which specifically detect both full-length TSG101 and the shorter splice variant TSG∆154-1054 (Figure 5E).

These results together indicate that TSG101 protein stabilization that is caused by TSG∆154-1054 is important to mediate tumor cell metastasis.

### 2.5. TSG∆154-1054 Expression Correlates with Tumor Metastasis in Patients of NPC

The demographics of our NPC patient cohort are shown in Table 1. Our NPC clinical outcome analysis revealed that the expression of TSG∆154-1054 is associated with early metastasis with statistical significance (chi-square test; *p* = 0.045), although the TSG∆154-1054 expression is only marginally correlated with advanced tumor stage (chi-square test; *p* = 0.077).

## 3. Discussion

When the human *TSG101* gene was first identified, genomic mutations and alternatively spliced mRNA isoforms were detected in human breast cancer [17]. Since then, splice variants of TSG101, particularly the major isoform, TSG∆154-1054, have been reported in many cancers, including breast, ovarian, lung, prostate, cervical, liver cancers, and leukemia [18,19,20,21,22,24]. Consistent with these findings, our data also illustrated that most NPC specimens from patients selectively express TSG∆154-1054, which is notably observed in all cases developing metastasis (NPC2, 22, 26 and 30 in Figure 1). Although the biological and medical significance of TSG∆154-1054 is strongly suggested by its association with tumor progression [24,30], the detailed mechanism of action for the TSG∆154-1054-mediated tumor progression is still poorly understood. Here, we provided evidence for the key role of TSG∆154-1054 in NPC progression through the stabilization of TSG101 protein.

We previously demonstrated that the generation of TSG∆154-1054 is a consequence of tumorigenic activation of a re-splicing of TSG101 mature full-length mRNA, and thus the completion of the normal splicing pathway activates the distant weak alternative splice sites [29]. Interestingly, it was shown that the cancer-specific splicing variant, TSG∆154-1054, is preferentially accumulated in TP53 (p53)-null cancer cell lines [28]. Moreover, the activation of TP53 by γ-irradiation reduces the expression of this TSG∆154-1054 variant [28], implying that the cancer-specific TSG101 aberrant splicing event is under the control of TP53. We are currently screening a siRNA library against human nuclear proteins to identify the direct repressors and activators of TSG101 mRNA re-splicing.

Using NPC biopsies, we first observed that the generation of aberrant TSG∆154-1054 mRNA correlates with increasing amounts of the full-length TSG101 protein. This observation was recapitulated in transiently and stably transfected cancer cells, in which TSG∆154-1054 expression indeed increases full-length protein, but not mRNA levels, of TSG101. This effect could be inhibited by the specific degradation of TSG∆154-1054 mRNA, indicating a direct correlation between the full-length TSG101 protein levels and the splicing variant TSG∆154-1054.

In mammalian cells, the amount of TSG101 protein is strictly counterbalanced by a post-translational process involving its ubiquitin-proteasome proteolysis [32,33]. A conserved RING E3 ubiquitin ligase, TSG101-associated ligase (Tal), has been shown to reduce TSG101 abundance by targeting TSG101 for ubiquitination, followed by its consequent degradation [33,34]. MDM2, another E3 ubiquitin ligase, also accelerates the turnover of excess TSG101 and acts with TSG101 in a regulatory loop of the MDM2-TP53 circuit [32]. Furthermore, the post-translational autoregulation of TSG101 has been shown to control the total intracellular pool of TSG101 and limit the protein to a steady-state level [35].

Despite these alternative degradation routes in controlling the TSG101 amount, increased levels of the TSG101 protein are nevertheless detected in a variety of cancers [36,37,38]. This discrepancy is likely attributable to the presence of TSG∆154-1054 in these cancer tissues. We previously demonstrated that the protein product of TSG∆154-1054 competitively binds to Tal, but not MDM2, thereby impeding the proper Tal binding to TSG101, leading to the protection of TSG101 from the subsequent polyubiquitination and following proteasomal degradation [31]. Our cycloheximide chase analysis indeed substantiated this TSG∆154-1054 triggered mechanism to protect the integrity of TSG101. Moreover, the upregulated TSG101 protein in TSG∆154-1054-positive NPC tissues provides clinical evidence to support a role of TSG∆154-1054 in promoting the stabilization of the TSG101 protein.

Conditional *Tsg101*-knockout mice revealed that the TSG101 protein is essential in cell proliferation and survival [9,10]. Regarding its mechanism of action in cell proliferation, it was shown that the TSG101 protein is recruited to the midbody during cytokinesis by interaction with centrosome protein 55 (Cep55), which is essential for abscission, the final stage of cytokinesis [39]. In ovarian cancer cells, increased TSG101 levels upregulate CBP/p300-interacting transactivator with ED-rich tail 2 (CITED2) and hypoxia-inducible factor 1α (HIF-1α), while downregulating tumor suppressor p21 and subsequently promoting cell growth and survival [37,40].

It was reported that siRNA-mediated downregulation of TSG101 caused partial cell cycle arrest and reduced the colony formation capacities of prostate and breast cancer cells, and notably, it diminished the migratory activity of breast cancer cells [11]. A recent study suggested that TSG101 promotes the proliferation, migration, and invasion of hepatocellular carcinoma cells by regulating the expression of *PEG10* (paternally expressed 10) gene [41]. Here, we demonstrated that cancer-specific byproduct TSG∆154-1054 stabilizes TSG101 protein, and thus it promotes tumor cell aggression in terms of proliferative, migratory, and metastatic activities. Recent TSG101 knockdown experiments implicated the function of TSG101 protein in anoikis (cell-detachment-induced apoptosis) resistance of thyroid cancer, which is a prerequisite for metastasis [42]. It is of interest to examine the activation of anoikis in TSGΔ154-1054-siRNA transfected cells in which TSG101 protein is upregulated. Since we found no activation of caspase 8 in these knockdown cells, a proapoptotic protein (BCL-2 like protein 4) and an apoptotic marker (cleaved poly-ADP ribose polymerase) may function in anoikis [42] through caspase 8-independent pathways.

Our results first highlighted the impact of TSG∆154-1054-mediated stabilization in the functional activities of TSG101. Moreover, subcutaneous inoculation of a cell line stably expressing TSG∆154-1054 into athymic nude mice substantially increases the efficiency of tumor formation *in vivo* [13]. This role of the splice variant is corroborated by the clinical observation of higher prevalence of metastasis in TSG∆154-1054-positive cancer patients. The importance of this cancer-specific splicing variant in NPC may be underestimated due to the limited number of studied patients to date.

In summary, we provide novel evidence of the TSG∆154-1054 protein function in regulating TSG101 stability during NPC tumorigenesis. TSG∆154-1054 plays a key role in promoting the metastasis of NPC *via* the stabilization of TSG101, which has recently been suggested to be used as a cancer biomarker [41,43,44]. Therefore, TSG∆154-1054 is a potential diagnostic biomarker and therapeutic target for cancer.

## 4. Materials and Methods

### 4.1. Primary Specimens from NPC Patients and Cell Lines

Thirty-eight NPC biopsies were used, with the primary tumors staged from I to IV, classified according to American Joint Committee on Cancer (AJCC) staging system. Thirty lymphoid hyperplasia (LH) biopsies from nasopharynx that had been pathologically diagnosed with an absence of cancer cells were recruited as the control group. The Departments of Otolaryngology and Pathology of National Taiwan University Hospital provided all of these fresh frozen-sectioned specimens. All of the procedures were conducted in accordance with the ethical standards of the committee on human experimentation of the College of Medicine, National Taiwan University (Institutional Review Board approval number: 201311004RIND). TSG∆154-1054 expressing TW01 stable lines were previously established in our laboratory [31].

TW01 cells, which are a human NPC cell line, were grown in Dulbecco’s modified Eagle’s medium (DMEM; Hyclone classical media, GE Healthcare Life Sciences, Marlborough, MA, USA), supplemented with 10% fetal calf serum (FCS; Hyclone classical media, GE Healthcare Life Sciences), L-glutamine, and penicillin-streptomycin. TW01 cells stably expressing TSGΔ154-1054 and the control empty vector (pcDNA3.1) were established in complete DMEM under G418-selection.

### 4.2. Construction of Plasmids and siRNAs

The expression plasmids encoding TSG101 and TSG∆154-1054 were generated by the PCR technique and were subcloned into the pcDNA3.1 vector (Invitrogen, Carlsbad, CA, USA). To yield fusion proteins, TSG101 was fused either with an N-terminal HA- or FLAG-tag. The construction of the pSuper-siE7 plasmid was described in our previous publication [13]. The siTSG∆154-1054-expressing plasmid was constructed by subcloning a short overlapping oligonucleotide as siRNA template into the pSUPER vector (Oligoengine, Seattle, WA, USA).

The siTSG∆154-1054 template (5′-GACCTAACTCTCCCTTATA-3′) is designed complementary to the TSG101 sequence nucleotides 145 to 153 (GACCTAACT) joined to nucleotides 1055 to 1064 (CTCCCTTATA). ON-TARGETplus SMARTpool siRNAs against human TSG101 (siTSG101, L-003549-00-0005) and non-targeting siRNA (siCtrl, D-001810-01-20) were purchased from GE Healthcare Life Sciences.

### 4.3. Transfection of Cells and siRNA Knockdown Experiments

Transfection of expression plasmids and ON-TARGETplus SMARTpool siRNAs was carried out using TransFast Transfection reagent (Thermo Fisher Scientific, Waltham, MA, USA) and DharmaFECT (GE Healthcare Life Sciences), respectively, following the manufacturers’ instructions. siRNAs were transfected twice into TW01 cells and, at 48 h post-transfection, transiently or stably transfected culture cells were harvested for the following experiments.

### 4.4. RNA Extraction, Reverse Transcription, and PCR Analysis

Total RNAs were isolated from cultured cells with TRIzol reagent (Thermo Fisher Scientific), as described by the manufacturer. Complementary DNA was generated from total RNAs and with randam hexmers and SuperScript III reverse transcriptase (Thermo Fisher Scientific), according to the manufacturer’s instructions. Nested-PCR analyses targeting TSG101 transcripts were carried out as previously described, with the primers P1 (5′-CGGTGTCGGAGAGCCAGCTCAAGAAA-3′) and P2 (5′-CCTCCAGCTGGTATCAGAGAAGTCAGT-3′) for the first-round PCR, and P3 (5′-AGCCAGCT CAAGAAAATGGTGTCCAAG-3′) and P4 (5′-TCACTGAGACCGGCAGTCTTTCTTGCTT-3′) for the second-round PCR [13,17]. To verify the ectopic expression of TSG∆154-1054, primers P3 and P4 were used in 30 cycles of PCR amplification at 95 °C for 50 s, 67 °C for 30 s, and 72 °C for 1 min. The internal control PCR in detecting the Defender Against Death-1 (DAD-1) was carried out as previously described [13]. The PCR products were analyzed by 1% agarose gel electrophoresis, followed by ethidium bromide staining. The relative intensity of the TSG∆154-1054 mRNA variant was quantified by densitometry ImageQuant software (GE Healthcare Life Sciences).

### 4.5. Immunoblot Analysis

48 h post-transfection, transiently or stably transfected culture cells were harvested and cell lysates were prepared using lysis buffer containing 3% SDS, 2 M urea, and 2% 2-mercaptoethanol. Immunoblotting was performed with antibodies directed against TSG101 (GeneTex, Irvine, CA, USA), HA (BABCO International, Tucson, AZ, USA), and GAPDH (Biodesign, Carmel, NY, USA), as described previously [13]. Expressed proteins were quantified by densitometry with ImageQuant software (GE Healthcare Life Sciences).

### 4.6. Cycloheximide Chase Assay for TSG101 Protein Stability

TW01 cells that were seeded onto six-well plate were incubated with cycloheximide (300 μg/mL) at 18 h post-transfection prior to harvesting at serial time points. The stability of HA-tagged TSG101 (HA-TSG101) was examined by immunoblotting and quantified by densitometry (see above). The cycloheximide chase assay was also performed with TSG∆154-1054 expressing TW01 stable cell lines [13], which were transfected twice with the siTSG∆154-1054 and once with HA-TSG101 expression plasmid.

### 4.7. Scratch-Wound Assay for Cell Migration Ability

Transfected TW01 confluent cells were maintained in DMEM containing 1% FCS and they were scratched using a sterilized micropipette tip. The monolayer cells were washed extensively with DMEM and then cultured in DMEM with 1% FCS for another 20 h. The migration of TW01 cells was observed under a bright-field microscopy (Axiovert, Zeiss). The migration rate (%) is calculated as (*g*_0h_ − *g*_20h_)/*g*_0h_, where *g*_0h_ and *g*_20h_ are the inter-scratch distances at time 0 h and 20 h, respectively.

### 4.8. Transwell Cell Invation Assay

24-well transwell chambers with 8 μm pore-size PET membranes (Corning, New York, NY, USA) were covered with 50 μL of Matrigel (Corning) and then incubated at 37 °C for 2 h. Transfected TW01 cells were cultured for 24 h, 2.5 × 10^4^ of these cells were suspended in 100 μL DMEM with 0.1% FCS, and then seeded onto the upper chambers of Matrigel-coated transwell plates. Subsequently, 500 μL of complete DMEM containing 10% FCS was added to the lower chamber. After 48 h, the non-migrating cells were removed from the upper chamber with a cotton swab. Cells that invaded into the bottom of the transwell membranes were fixed with 4% paraformaldehyde for 10 min at room temperature, stained with KaryoMax Giemsa Stain solution (Thermo Fisher Scientific), and the stained cells were counted. Quantification of the invasion ability was determined by the invaded cell number.

### 4.9. Statistical Analysis

Aberrantly spliced TSG101 products in cancer and control tissues were assessed while using the chi-square test and Mann–Whitney (Wilcoxon) rank sum test. In the scratch-wound assay to test cell migration activity, each image was a representative result from three independent experiments that were performed in duplicate and the effect of TSG∆154-1054 was evaluated by a two-tailed *t*-test. The correlations between TSG∆154-1054 positivity and TNM stage or metastasis were assessed by the Chi-square test.

## Figures and Tables

**Table 1:** Patient demographics.

Characteristics	No. of Patients		*p* Value **
	TSG ∆154-1054 (+) *	TSG ∆154-1054 (−)	
**Age**			
Median (Extent)	44.8 (32–62)	47.3 (24–78)	
**Gender**			0.825
Male	12	14	
Female	6	6	
**Pathology Subtype**			0.840
Non-keratinizing	4	5	
Undifferentiated	14	15	
**Primary Tumor Stage (T)**			0.165
T1	2	2	
T2	3	8	
T3	3	1	
T4	9	4	
**Nodal Stage (N)**			0.287
N0	3	5	
N1	4	6	
N2	7	2	
N3	3	2	
**Metastasis**	4	0	0.045
Stage ***			0.077
Early (I + II)	3	7	
Late (III + IV)	14	8	
**Total Number of Patients**	18	20	

* Cases that detected positive for TSG∆154-1054 was ascertained twice in two independent RT-nested-PCR experiments performed in duplicate. ** Chi-square test was performed to evaluate the correlation between TSG∆154-1054 positivity and clinical outcomes. *** Missing data in one case for TSG∆154-1054 (+) and in five cases for TSG∆154-1054 (−).

## Abbreviations

*TSG101*	*Tumor susceptibility 101* gene
NPC	Nasopharyngeal carcinoma
ESCRT	Endosomal sorting complex required for transport
PCR	Polymerase chain reaction
DMEM	Dulbecco’s modified Eagle’s medium
FCS	Fetal calf serum
